# Rapid Least Concern: towards automating Red List assessments

**DOI:** 10.3897/BDJ.8.e47018

**Published:** 2020-01-23

**Authors:** Steven Bachman, Barnaby Eliot Walker, Sara Barrios, Alison Copeland, Justin Moat

**Affiliations:** 1 Royal Botanic Gardens, Kew, London, United Kingdom Royal Botanic Gardens, Kew London United Kingdom; 2 Department of Environment and Natural Resources, Government of Bermuda, Hamilton, Bermuda Department of Environment and Natural Resources, Government of Bermuda Hamilton Bermuda

**Keywords:** Red List, Least Concern, Non-threatened, plants, automation, GBIF, Plants of the World Online, Bermuda

## Abstract

**Background:**

The IUCN Red List of Threatened Species^TM^ (hereafter the Red List) is an important global resource for conservation that supports conservation planning, safeguarding critical habitat and monitoring biodiversity change (Rodrigues et al. 2006). However, a major shortcoming of the Red List is that most of the world's described species have not yet been assessed and published on the Red List ([Bibr B5202506][Bibr B5272167]). Conservation efforts can be better supported if the Red List is expanded to achieve greater coverage of mega-diverse groups of organisms such as plants, fungi and invertebrates. There is, therefore, an urgent need to speed up the Red List assessment and documentation workflow.

One reason for this lack of species coverage is that a manual and relatively time-consuming procedure is usually employed to assess and document species. A recent update of Red List documentation standards (IUCN 2013) reduced the data requirements for publishing non-threatened or 'Least Concern' species on the Red List. The majority of the required fields for Least Concern plant species can be found in existing open-access data sources or can be easily calculated. There is an opportunity to consolidate these data and analyses into a simple application to fast-track the publication of Least Concern assessments for plants. There could be as many as 250,000 species of plants (60%) likely to be categorised as Least Concern (Bachman et al. 2019), for which automatically generated assessments could considerably reduce the outlay of time and valuable resources for Red Listing, allowing attention and resources to be dedicated to the assessment of those species most likely to be threatened.

**New information:**

We present a web application, **Rapid Least Concern**, that addresses the challenge of accelerating the generation and documentation of Least Concern Red List assessments. **Rapid Least Concern** utilises open-source datasets, such as the Global Biodiversity Information Facility (GBIF) and Plants of the World Online (POWO) through a simple web interface. Initially, the application is intended for use on plants, but it could be extended to other groups, depending on the availability of equivalent datasets for these groups.

**Rapid Least Concern** users can assess a single species or upload a list of species that are assessed in a batch operation. The batch operation can either utilise georeferenced occurrence data from GBIF or occurrence data provided by the user. The output includes a series of CSV files and a point map file that meet the minimum data requirements for a Least Concern Red List assessment (IUCN 2013). The CSV files are compliant with the IUCN Red List SIS Connect system that transfers the data files to the IUCN database and, pending quality control checks and review, publication on the Red List.

We outline the knowledge gap this application aims to fill and describe how the application works. We demonstrate a use-case for **Rapid Least Concern** as part of an ongoing initiative to complete a global Red List assessment of all native species for the United Kingdom Overseas Territory of Bermuda.

## Introduction

The global Red List is incomplete, with plants, fungi and invertebrates representing the major gaps in coverage. Despite major efforts to document the Red List status of plants (Brummitt et al. 2015, Raimondo et al. 2013, Rivers 2017), only ~10% of species have assessments published on the Red List so far (IUCN 2019). Without comprehensive coverage of species on the Red List, we limit our ability to utilise the Red List as a tool for conservation.

To speed up the assessment process, we can adopt a two-stage strategy: prioritisation and automation. Prioritisation is necessary because ongoing and intensifying threats to biodiversity (Symes et al. 2018, Venter et al. 2016) and limited resources (McCarthy et al. 2012) necessitate a rapid response. The current Red List of plants (IUCN 2019) indicates that some form of prioritisation has already been adopted because the proportion of plant assessments in the threatened categories (Critically Endangered, Endangered and Vulnerable, 43%) is higher than the estimated global proportion of threatened plant species (21%, Brummitt et al. 2015). However, there is still a vast number of species to assess, with as many as 115,000 species (27%) estimated to be of elevated conservation concern (species classified as threatened or in the Near Threatened category) and ~250,000 (60%) estimated to be in the Least Concern category (Bachman et al. 2019). The assessment and documentation of threatened species for the Red List requires training, experience and careful interpretation of the Red List guidelines to ensure high quality assessments are generated (Hayward et al. 2015). There is a growing, but still limited, pool of plant Red List assessors that work with the IUCN Species Survival Commission (SSC) Plant Specialist Groups and IUCN Red List partners to generate assessments. It is therefore vital that this highly valuable assessor network is applied to the task of assessing species most likely to be threatened, rather than expending effort on the lower risk 'Least Concern' species. An approach to rapid assessment of Least Concern species from open-source datasets, followed by prioritisation of threatened species, has already been adopted to great effect by the Global Tree Assessment (GTA) (Rivers 2017).

To ensure that the assessor network can focus primarily on threatened species, we need user-friendly tools to both prioritise and automate the assessment of the remaining pool of 'Not Evaluated' plant species. The first step is to apply a triage approach that classifies species into those likely to be threatened and those likely non-threatened. Secondly, for the non-threatened species, the assessments should be automatically generated.

**Prioritisation**:

Several approaches to prioritise plant species for assessments have shown a high level of accuracy (> 96%) when predicting non-threatened species, especially those that incorporate some measure of geographic range (see Nic Lughadha et al. (2018) for a recent review). However, these approaches often assume a clean dataset of georeferenced occurrence points already exists from which predictive models can be generated or spatial metrics calculated. Despite some gaps in coverage (Meyer et al. 2015), data aggregators, such as the Global Biodiversity Information Facility (GBIF, www.gbif.org), have aggregated considerable amounts of occurrence data that can be harnessed for estimating the geographic range. However, a shortcoming of the data is the lack of distinction between native and non-native occurrences. Uncritical use of these data can lead to overestimation of geographic metrics, such as extent of occurrence (EOO) and area of occupancy (AOO), as used in tools such as GeoCAT (Bachman et al. 2011), potentially leading to a mis-classification of the Red List category. An ongoing project to develop a checklist of all plants (Plants of the World Online (POWO), http://plantsoftheworldonline.org/) utilises the World Geographical Scheme for Recording Plant Distributions to document native and introduced geographic ranges for species at a relatively coarse scale. By using native ranges from POWO as a cleaning filter, we can reduce the risk that geographic metrics from occurrence records are overestimated, therefore avoiding mis-classification of potentially threatened species as non-threatened. The resulting list of potentially threatened species can then be prioritised for full asessment and the non-threatened species can be automatically documented to meet IUCN Red List standards.

**Automatic generation of Least Concern assessments**:

The second stage is to fully document those species identified as Least Concern. All Red List assessments require three elements: the assessment category, supporting documentation and a distribution map (IUCN 2013). For Least Concern species, the required supporting data include the scientific name along with higher taxonomic details and taxonomic authority, Red List Category, rationale, countries of occurrence, population trend, habitats, ecological system and bibliography. Each of these requirements can be met using default values (e.g. 'LC' for the Red List category) or calculated using data from GBIF and Plants of the World Online. The raw occurrence data form the basis of the distribution map and can be modified to conform to the IUCN Mapping Standards (Red List Technical Working Group 2018).

**SIS Connect - bridging raw data and the Red List**

All published assessments on the Red List are drawn from an underlying database called the Species Information Service. Until recently, supporting documentation for Red List assessments had to be entered into SIS manually. The recent development of **SIS Connect** (http://connect.iucnredlist.org/) has enabled batch transfer of assessments to SIS. Batch transfer requires preparation of a compressed file containing multiple CSV files that collectively make up the raw data of Red List assessments. After registering and logging in to SIS Connect, the user can upload the compressed file, which is then subject to validation checks. If approved by the Red List Unit and subsequently reviewed by the relevant Red List Authority, the assessments represented in the batch file can be processed for publication on the Red List.

## Project description

### Title

Rapid documentation of Least Concern plants for the Red List

### Study area description

Can be applied worldwide across all plants. Case study from Bermuda.

### Design description

The **Rapid Least Concern** user interface can be accessed from the following link: https://spbachman.shinyapps.io/rapidLC. From the home page, the user can choose the single or batch species workflow, access the quick-start video tutorials or access the user manual via the help tab (Suppl. material 1).

**Figure 1.**
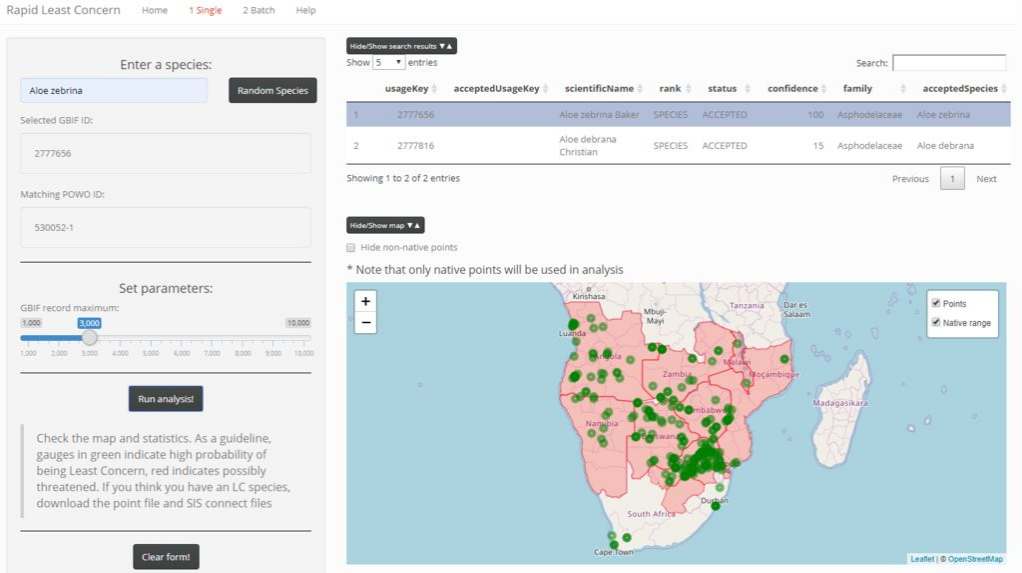
Screenshot of single assessment workflow using *Aloe zebrina* Baker as an example species.

**Single species workflow**:

The single species workflow requires the user to enter a plant species name in the binomial form i.e. *Genus species* into the text input box on the left side panel (Fig. 1). A fuzzy search of the GBIF name backbone is initiated via the GBIF Species API using *rgbif* (Chamberlain et al. 2019a) and results are displayed in a table in the main panel to the right. Taxonomic details, including family, author and level of confidence in the matching name, are reported, with the best match at the top of the list. The user can then select the most appropriate match, which initiates a search of Plants of the World Online (POWO). We used the *httr* package (Wickham 2018) to request data from POWO and *jsonlite* (Ooms 2014) to parse the data returned from requests. As the POWO API has not yet been published, a python library has been developed as the recommended access point for POWO services and an integration has been made with the R taxize package (Chamberlain et al. 2019). A matching name in POWO can then be used to access the species distribution range, according to the Taxonomic Database Working Group (TDWG) World Geographical Scheme for Recording Plant Distributions (WGSRPD). POWO records native and introduced ranges at Level 3 of the WGSRPD, which is equivalent to small to medium sized countries.

The GBIF Occurrence API is then accessed via the GBIF usageKey using the selected plant name to query all georeferenced occurrence records that do not have a geospatial issue. A parameter to determine the upper limit for the number of occurrences can be set using the slider widget where a minimum of 1,000 and maximum of 10,000 occurrences are permitted with a default of 3,000 occurrences. Clicking the 'Run analysis!' button will initiate a spatial query of the occurrence records within the native range, such that all non-native occurrence records are excluded from any further analysis. The occurrence records and native range are visualised on a base map (Fig. 1). The following statistics, extent of occurrence (EOO), area of occupancy (AOO), number of occurrence records and number of TDWG Level 3 regions, are generated from the native range occurrence points (Table 1) and are used to determine whether a species is likely to be Least Concern. A visualisation to assist with interpretation is provided with a series of gauges for each of the four parameters, EOO, AOO, RecordCount and TDWGCount, that are highlighted in green if Least Concern thresholds are met or exceeded, or red if below thresholds. This ends the prioritisation stage and the user can now decide whether to accept the suggested rating or not.

It should be noted that the application primarily uses range-based parameters to determine Least Concern status, which, although shown to be good predictors of threat status (Nic Lughadha et al. 2018, Darrah et al. 2017), ignore other factors that could make a widespread species eligible for a threatened or near threatened category, such as disease, deforestation or over-exploitation that affect the entire range. Users must consider any observed, estimated, projected, inferred or suspected declines likely to trigger Red List criteria A, B, C, D or E, in line with IUCN Red List guidelines (IUCN Standards and Petitions Subcommittee 2017).

The 'Run analysis!' button also initiates the generation of several data tables that form the basis of the minimum required documentation for a Least Concern assessment (IUCN 2013). The user can download a CSV file for the point distribution, as well as a zip file containing the following CSV files: *allfields*, *assessments*, *countries*, *credits*, *habitats*, *plantspecific*, *taxonomy* that are all linked through the *internal_taxon_id* field. Registered users can then upload the CSV files to the SIS Connect website to enable transfer to the IUCN SIS database.

**Batch species workflow**:

The batch species workflow offers the option to apply the single species workflow to multiple species at once. Instead of entering a name, the user can upload a CSV file containing multiple names. The batch analysis runs an initial test of the name against POWO and returns a report on the number of name matches or names recognised by POWO as synonyms. Any names not matched to POWO or listed as synonyms are excluded from further analysis. Again, the parameter of GBIF occurrence record limit can be set and then the analysis returns a table of results comprising the same statistics as for the single species workflow: extent of occurrence (EOO), area of occupancy (AOO), number of occurrence records and number of TDWG Level 3 regions for each species.

Unlike the single workflow, the user can adjust the LC thresholds using the sliders on the left-hand side bar which will refresh the table of results. The user can then download the compressed folder containing CSV files for all species that met or exceeded the LC thresholds. In most tables, a separate row is added for each species, except for tables where there is a one-to-many relationship, (e.g. habitat), where a species can occur in multiple habitat types and each is recorded in a separate row. A results CSV file is also included in the download and includes the raw statistics for each species, whether or not it was LC, according to the thresholds and a note in the 'warning' column to explain any issues, for example, there were no georeferenced points from GBIF to carry out the area calculations.

An extension of the batch workflow allows users to upload their own occurrence data as they may already have a cleaned dataset prepared. The process is exactly the same as above, except the user uploads a CSV file with names alongside decimal latitude and longitude co-ordinates. In this scenario, the GBIF occurrence download is bypassed and the user occurrence points are used to calculate the spatial metrics.

**Threshold values**:

Although there are no defined thresholds to separate Near Threatened species from Least Concern species, the Red List guidelines indicate that an extent of occurrence (EOO) > 30,000 km^2^ and AOO > 3,000 km^2^ are not likely to trigger a threatened or Near Threatened category and hence can be classified as Least Concern (IUCN Standards and Petitions Subcommittee 2017). The reference scale for calculating area of occupancy (AOO) for IUCN Red List assessments is 2 km by 2 km (4 km^2^). Estimation of AOO from occurrence data is problematic due to variation in sampling effort, often leading to underestimation of AOO (IUCN Standards and Petitions Subcommittee 2017). In addition, the majority of raw occurrence data from GBIF has an unquanitified level of georeference accuracy. To account for a potential lack of precision, the AOO metric is measured at a broader scale of 10 km by 10 km (100 km^2^). This coarse scale-estimate of AOO is only being used to help determine whether species are likely to be Least Concern or not; it is not a valid estimate of AOO in the strict sense of IUCN at the 2 km by 2 km (4 km^2^) scale. For the specimen record count, previous work has shown that high accuracy (82 - 90%) in predicting threat status can be achieved with < 15 specimen records (Nic Lughadha et al. 2018). However, the rapid growth of digitally accessible information (DAI) may make it increasingly likely that a potentially restricted and threatened species could be represented by > 15 specimens simply due to availability of multiple samples from the same population or duplicated records. Therefore, this threshold cannot be relied upon in the longer term.

**Figure 2.**
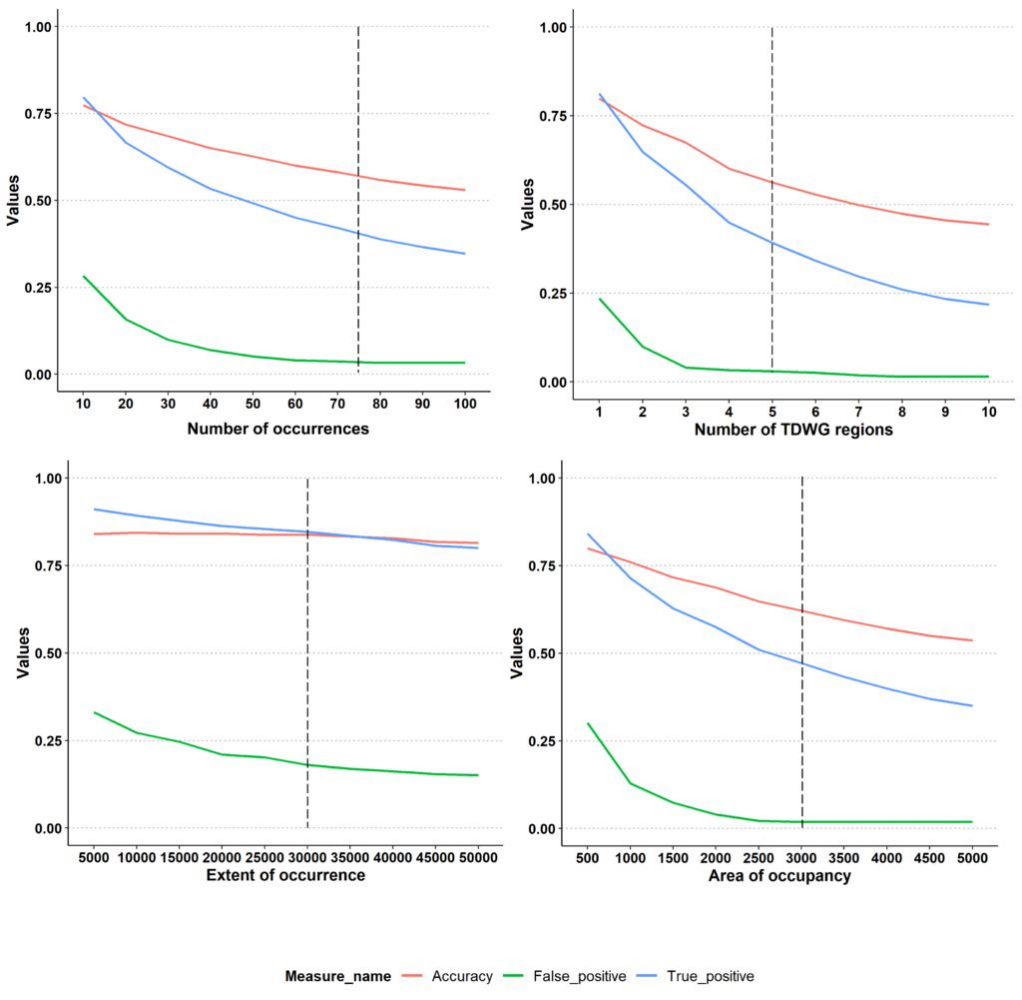
Sensitivity analysis to determine thresholds for Least Concern for each parameter: number of specimens, number of TDWG regions, extent of occurrence and area of occupancy. Vertical dashed line shows the chosen threshold (also reported in Table 1). We used 923 randomly selected monocot species from the Sampled Red List Index for Plants Project (Brummitt et al. 2015) as the validation dataset. For each species, we compared the published Red List assessments with those predicted by Rapid Least Concern over a continuum of threshold values and reported accuracy measures based on a confusion matrix.

In order to determine an appropriate threshold for each parameter, we carried out a sensitivity analysis using, as a validation dataset, a random set of 923 monocot species with complete, published Red List assessments from the Sampled Red List Index for Plants Project (Brummitt et al. 2015). For each species, we compared the published Red List assessments with those predicted by Rapid Least Concern over a continuum of threshold values and reported accuracy measures based on a confusion matrix. Overall, the accuracy for each parameter declines as the thresholds are increased (Fig. 2). As the aim of the application is to identify Least Concern species, it is necessary to minimise the number of false positives, i.e. predicting Least Concern when the species is threatened, whilst maximising true positives, i.e. predicting Least Concern when it is Least Concern. Even at the highest tested thresholds, we did not achieve a zero false positive rate. For example, *Ansellia africana* Lindl. has a large range, represented by many occurrence records in GBIF and was predicted to be Least Concern in all threshold scenarios, but the published Red List rating is Vulnerable (Crook 2013). The threatened status of *Ansellia africana* Lindl. is due to over-harvesting across the range. To account for such situations, prior to downloading results, we ask users to consider for each species any observed, estimated, projected, inferred or suspected declines likely to trigger Red List criteria A, B, C, D or E, in line with IUCN Red List guidelines (IUCN Standards and Petitions Subcommittee 2017). In addition, each Least Concern assessment should be made available for review by all relevant Red List Authorities and any reviewers' comments addressed prior to publication on the Red List.

The chosen thresholds listed in Table 1 are intended to reflect the balance between correctly predicting Least Concern species, while reducing false positives. Considering our testing set of 923 monocots, there is scope to make minor reductions in the threholds for number of specimens, number of TDWG regions and area of occupancy to increase the number of true positives (LC species correctly predicted as LC) without substantially affecting the false positive rate. Our selected default threshold values are, therefore, in line with the precautionary principle as they seek to avoid falsely declaring a species as Least Concern, when it could be threatened; nonetheless, the user can adjust the thresholds according to expert knowledge of the group being assessed or the requirements of their project.

**Limitations**

The batch species workflow currently processes up to 100 species at a time. Processing time can vary depending on the species and mostly on the number of occurrences per species. Batches of more than 100 species are not recommended because performance issues are encountered when dealing with sets of more than 100 species in the IUCN central database (SIS).

The coverage and accuracy of the underlying datasets used by *Rapid Least Concern*, such as GBIF and Plants of the World Online, will influence the results generated. Despite known gaps in coverage and quality (Meyer et al. 2015), we have shown that these datasets can be successfully applied to the task of predicting Least Concern species. Continued efforts to mobilise and curate these data, for example, mass-digitisation and georeferencing or ensuring native distributions are accurately documented in Plants of the World Online, will improve the overall effectiveness of tools like *Rapid Least Concern*.

**Case study: Bermuda**

We illustrate the utility of *Rapid Least Concern* with a use-case for the plants of the UK Overseas Territory of Bermuda. Bermuda has a sub-tropical climate and a total land mass of just 53 km². Bermuda’s Department of Environment and Natural Resources and the UK Overseas Territories team at the Royal Botanic Gardens, Kew have been collaborating to assess the global Red List status of Bermuda’s native plant species. This collaboration has resulted in ten endemic plant species assessed and published on the IUCN Red List. To prioritise the assessment of the remaining native flora and to generate Least Concern Red List assessments for the non-threatened taxa at global level, we applied *Rapid Least Concern*.

We queried the Plants of the World Online database to obtain a checklist of all native plants for Bermuda and cross-checked with Britton's (Britton 1918) Flora of Bermuda, the UK Overseas Territories Online Herbarium (Hamilton and Barrios 2019) and the Bermuda Plant Finder (https://environment.bm/bermuda-plant-finder), published by the Department of Environment and Natural Resources of Bermuda. We also checked the IUCN Red List to see whether any other native Bermuda species had been assessed, in addition to the ten priority species. All plant species that were considered introduced or naturalised on Bermuda were removed from the combined list. The resulting final species list contained 172 plant species considered native to Bermuda, of which 38 (22%) had already been assessed for the Red List including nine (24%) that were listed as threatened (Suppl. material 2).

We used the *Rapid Least Concern* batch species workflow and uploaded a list of the remaining 134 Not Evaluated species. We used default values for the upper limit of GBIF occurrences (3,000) and default thresholds to determine Least Concern (Table 1).

**Figure 3.**
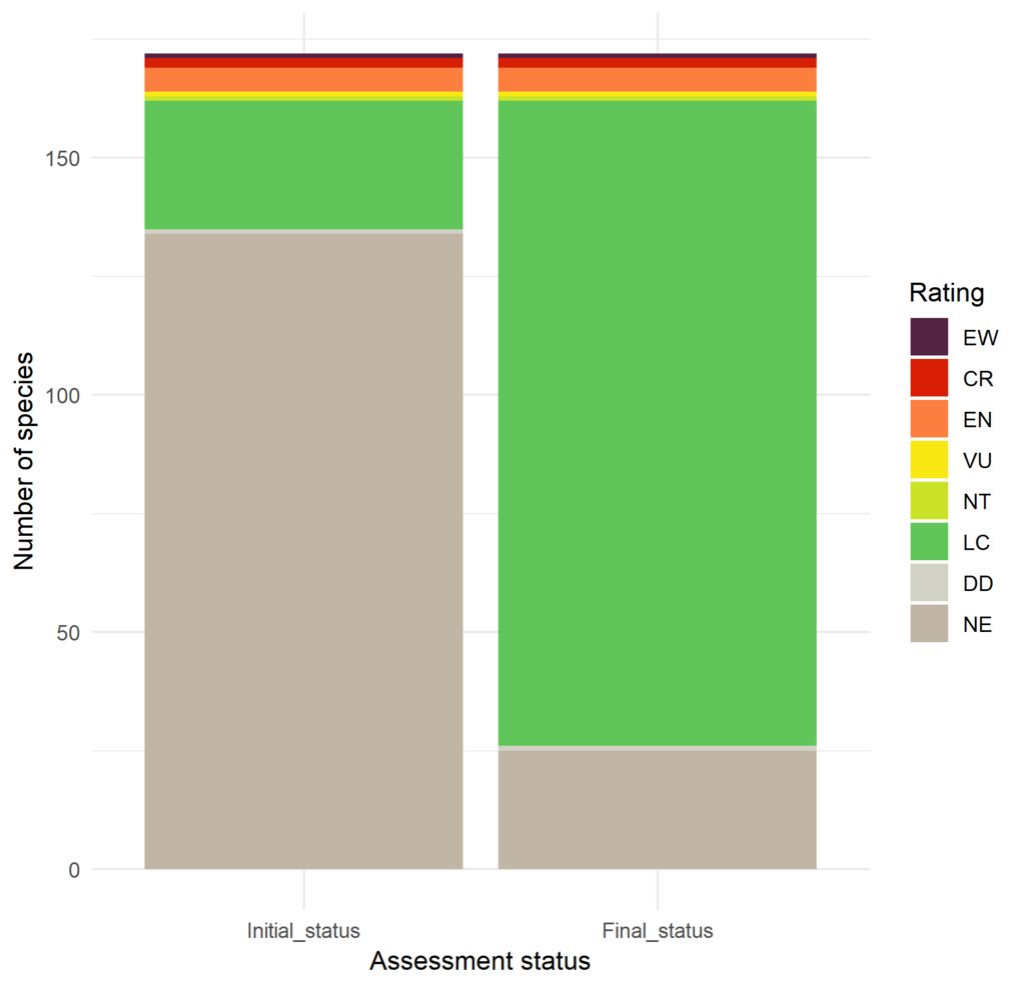
Impact of *Rapid Least Concern* on overall proportion of species assessed for native plants in Bermuda. Number of species in each Red List category are shown prior to and after running *Rapid Least Concern*. Categories follow the IUCN Red List system: Extinct in the Wild (EW), Critically Endangered (CR), Endangered (EN), Vulnerable (VU), Near threatened (NT), Least Concern (LC), Data Deficient (DD) and Not Evaluated (NE). All species have been assessed at global level.

**Outcomes**:

The overall proportion of species assessed at a global level increased from 38 (22%) to 147 (85%) (Fig. 3). From the list of 134 Not Evaluated species, *Rapid Least Concern* could not assign a value to ten species as there were insufficient data. For the remaining 124 species, 109 were assigned as Least Concern and 15 were assigned as possibly threatened. After a review of the proposed LC species, we agreed that all 109 species should be LC and the generated data files were sent to IUCN via SIS Connect. The remaining 25 species (15 listed as possibly threatened and 10 with insufficient data to assign a category) were considered as Not Evaluated and will be prioritised for full Red List assessments.

## Web location (URIs)

Homepage: https://github.com/stevenpbachman

## Technical specification

Platform: *shiny* for R

Programming language: R

Operational system: Windows, OSx, Linux

Interface language: English

## Repository

Type: Github

## Usage rights

### Use license

Other

### IP rights notes

MIT License

## Implementation

### Implements specification

*Rapid Least Concern* was written in the R programming language (R Core Team 2019). The code from the repository: https://github.com/stevenpbachman/rapidLC is implemented as a shiny web application using the *shiny* package (Chang et al. 2018) and is deployed to the shinyapps.io cloud service.

The application depends on the following R packages: *raster* (Hijmans 2019), *here* (Müller 2017), *magrittr* (Bache and Wickham 2014), *rgdal* (Bivand et al. 2019), *DT* (Xie et al. 2018), *leaflet* (Cheng et al. 2018), *rgbif* (Chamberlain et al. 2019a), *jsonlite* (Ooms 2014), *tidyverse* (Wickham 2017), *httr* (Wickham 2018), *zip* (Csárdi et al. 2019), *shinythemes* (Chang 2018), *wicket* (Keyes 2017), *sf* (Pebesma 2018), *rCAT* (Moat 2017), *flexdashboard* (Iannone et al. 2018), *shinydashboard* (Chang and Ribeiro 2018), *shinyjs* (Attali 2018).

### Audience

This application is targeted towards any user wishing to assess and document Least Concern assessments of plants for the Red List. It should be particularly useful for members of the IUCN Species Survival Commission (SSC) Plant Specialist Groups and IUCN Red List partners. It may also be of use to conservation practitioners wishing to quickly prioritise species for further detailed assessments or deprioritise those likely to be non-threatened. Finally, it can be used to rapidly generate baseline data for potentially threatened species.

## Supplementary Material

7395130B-D1B7-5683-BABA-932C3AC8C1A7 10.3897/BDJ.8.e47018.suppl1 Supplementary material 1 Rapid Least Concern - Help notes Data type: User manual
Brief description: Rapid Least Concern User Guide.
File: oo_327340.pdf https://binary.pensoft.net/file/327340 Steven Bachman

D066BAAC-3414-5BE5-9CE4-749C5FCBA8A5 10.3897/BDJ.8.e47018.suppl2 Supplementary material 2 Bermuda species list Data type: Species
Brief description: List of native plant species in Bermuda. Includes Red List status if already assessed, predicted Red List status (LC or not LC) and final category decided after expert review.
File: oo_344177.xlsx https://binary.pensoft.net/file/344177 Steven Bachman, Sara Barrios, Alison Copeland

## Figures and Tables

**Table 1.** Statistics used to determine Least Concern status with thresholds.

Field	Description	LC Threshold
EOO	Extent of occurrence. Calculated as the area (km^2^) of a minimum convex polygon of all extant occurrence points within the native range.	30,000 km^2^
AOO	Area (km^2^) calculated by summing the number of occupied cells based on occurrence points within the native range by the area of the cells. A grid of 10 km x 10 km cells was used to account for georeference error as opposed to the standard 2 x 2 km reference scale. A single occupied cell would return an AOO value of 100 km^2^	3,000 km^2^
RecordCount	The number of unique georeferenced occurrence records within the native range.	75
TDWGCount	The number of Level 3 TDWG regions in which the species occur across its native range.	5
POWO_ID	Unique ID for Plants of the World Online	
full_name	Binomial	
warning	Indicates a problem with analysis e.g. no points in GBIF	
leastConcern	Indicates whether the species meets or exceeds all the LC thresholds.	TRUE/FALSE
