# Supplementary material for: Rapid Least Concern: towards automating Red List assessments
Source: Biodivers Data J. 2020 Jan 23;8:e47018. doi: 10.3897/BDJ.8.e47018 (PMC6992691; doi:10.3897/BDJ.8.e47018)
Supplement: Supplementary material 1 — Rapid Least Concern - Help notes [file bdj-08-e47018-s001.pdf]

Rapid Least Concern - Help notes

Table of Contents

What is Rapid Least Concern? 1
Why create Rapid Least Concern? 1
Get involved: 2
How to use: 2
Single 2
Batch 7
Next steps: 10
Review assessments (SGs and RLAs) 10
Submit to SIS connect - needs login 10
Acknowledgements: 10

What is Rapid Least Concern?

Harnessing open-source data provided by GBIF - the Global Biodiversity Information Facility (GBIF) and Plants of the World (POWO), *Rapid Least Concern* carries out analysis of plant distributions to determine if they are likely to be threatened or not. For now, the threatened species will require further attention before publication on the Red List , but the non-threatened species require considerably less documentation and can be automatically generated. Rapid Least Concern provides both the analysis to determine if threat is likely and for the non-threatened species, also allows users to download the data in a format compliant with the IUCN Red List.

Why create Rapid Least Concern?

Quite simply, we want to speed up the rate at which assessments are generated for the IUCN Red List of Threatened Species. To date, only ~9% of plants have been assessed and ~250,000 species are estimated to be in the Least Concern category. A workflow developed for the Global Tree Assessment has proven that automation is possible and large volumes of Least Concern assessments of trees are already being transferred to the Red List. However, there are many more species to assess and there were no freely avaiable tools that apply a similar automation procedure. The develpment of Rapid Least Concern will speed up the process of documenting the un-assessed Least Concern plants will make a major contribution to the Red List and will mean that valuable assessor resources can be targeted towards assessing species most likely to be threatened.

Get involved:

You can help us improve Rapid Least Concern by letting us know of any bugs or by suggesting new features here:

How to use:

There are two options for generating LC assessments: **Single** and **Batch**. The single option first tests whether your species is likley to be Least Concern, and then allows you to download the data files needed to support publication of the assessment on the Red List. The batch option runs in the same way as the single, but allows users to process multiple species at the same time by uploading a csv. file with a list of names. You may also have a list of names with clean point data already associated with the names - in this case the batch process will run by using your points rather than searching GBIF for occurrence records.

Single

Try the quick start demo first:

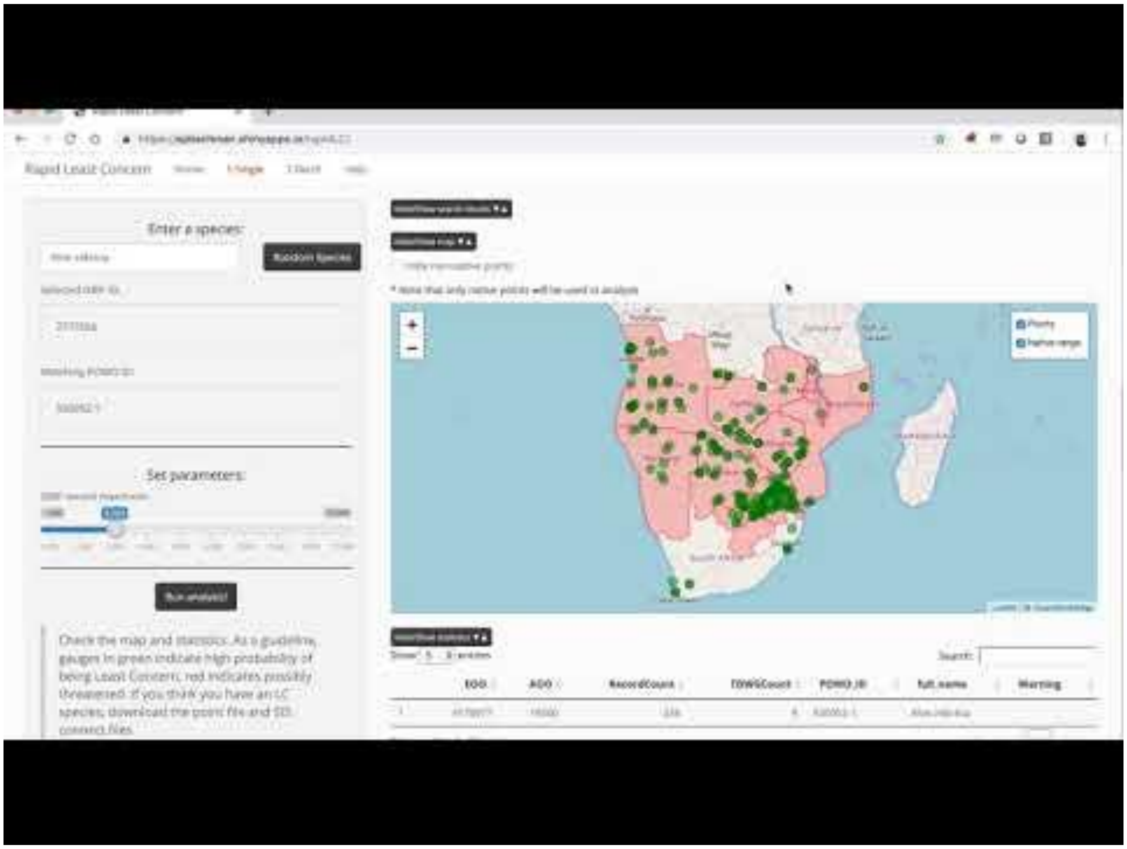

Step 1 - Enter a binomial (Genus species) into the 'Enter a species' search box. A table of results will appear in the main panel to the right. The results are from a search of the binomial against the Global Biodiversity Information Facility (GBIF) names backbone. The

best matches are listed in order of confidence. The *scientificName* field includes the author and can be used to make sure you find the species you are looking for.

Select a species from the table by clicking on a row, the row will be highlighted in blue. A match is then made to the Plants of the World (POWO) names backbone. Both the GBIF and POWO identifiers are reported in the left side bar. If there is no matching POWO identifier, the analysis cannot proceed.

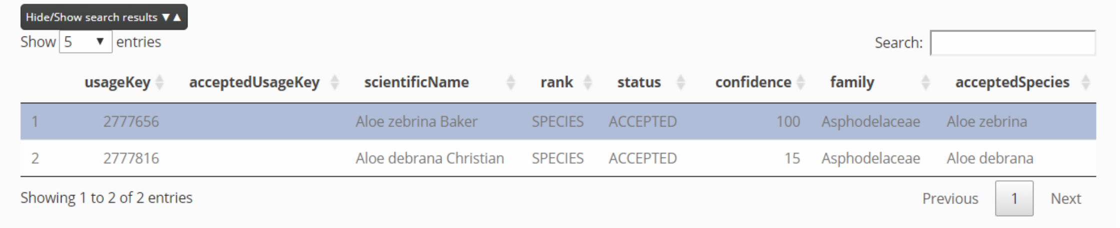
Hide/Show search results ▼▲

Show 5 entries | Search:

	usageKey	acceptedUsageKey	scientificName	rank	status	confidence	family	acceptedSpecies
1	2777656		Aloe zebrina Baker	SPECIES	ACCEPTED	100	Asphodelaceae	Aloe zebrina
2	2777816		Aloe debrana Christian	SPECIES	ACCEPTED	15	Asphodelaceae	Aloe debrana

Showing 1 to 2 of 2 entries | Previous 1 Next

Search results for Aloe zebrina

Step 2 - Set parameters A limit can be set to the number of occurrence records to be downloaded from GBIF. The value can be set with the slider widget. We allow a maximum of 10,000 occurrence points, a minimum of 1,000 and the default is set at 3,000.

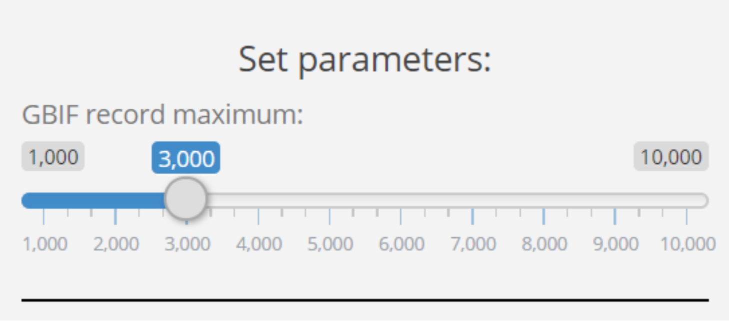

Set the maximum limit for GBIF occurrences

Step 3 Click the Run Analysis button. The first output is a map of the georeferenced occurrence points derived from GBIF (green circle markers), and the native range according to Plants of the World Online using the TDWG geographic distribution system (red polygons). The points and native range layers can be turned on or off and the non-native points can be hidden. Note that non-native points are not used in the analysis.

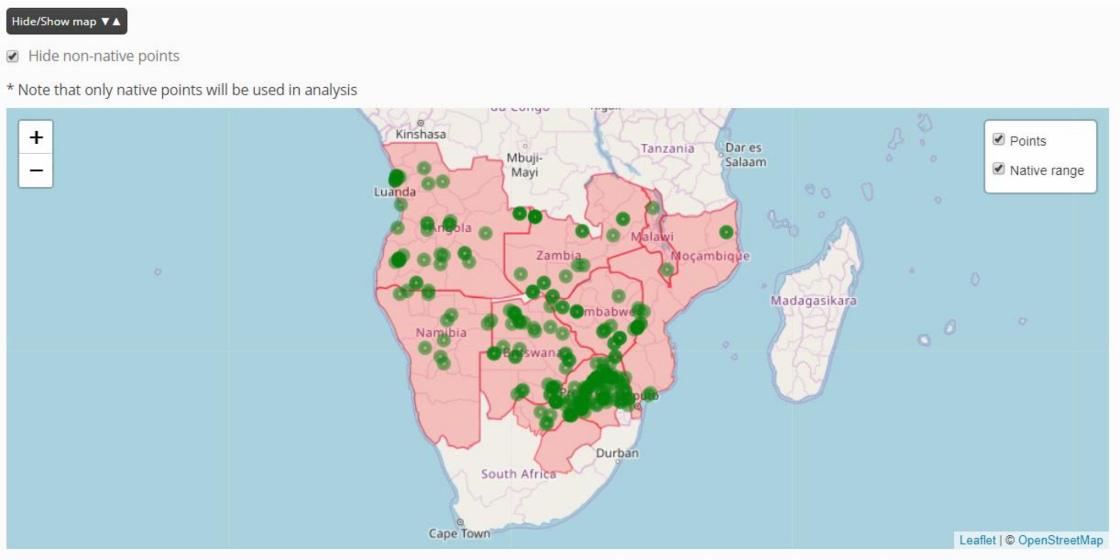

Distribution of Aloe zebrina

Statistics table and gauges. A table of statistics is loaded below the map with the following fields:

Field	Description	LC Threshold
EOO	Extent of occurrence. Calculated as the area (km^2) of a minimum convex polygon of all extant occurrence points within the native range.	30,000 km sq
AOO	Area (km^2) calculated by summing the number of occupied cells based on occurence points within the native range by the area of the cells. A grid of 10 km x 10 km cells was used to account for georeference error as opposed to the standard 2 x 2 km reference scale. A single occupied cell would return an AOO value of 100 km^2	3,000 km sq
RecordCount	The number of unique georeferenced occurrence records within the native range.	75
TDWGCount	The number of Level 3 TDWG regions the species occupies across its native range.	5
POWO_ID	Unique ID for Plants of the World Online	
full_name	Binomial	
warning	Indicates a problem with analysis e.g. no points in GBIF	

leastConcern	Indicates whether the species meets or exceeds all the LC thresholds. Values TRUE/FALSE

When the calculated values have met or exceeded the LC thresholds, the gauges turn green, indicating the species is not likely to be threatened. If the values are below the thresholds, the gauges will turn red, indicating the species may be threatened.

Note that currently the Rapid Least Concern app only considers range based metrics and number of occurrences as predictors of non-threatened status. Although these predictors are known to discriminate threatened and non-threatened status to high levels of acuracy (see Darrah *et al.* and Nic Lughadha *et al.* it is vital that the assessor considers other potential threatening processes before commiting to the generation of a Least Concern assessment. The user should consider if there are any observed, estimated, projected, inferred, or suspected declines likely to trigger criteria A, B, C, D or E.

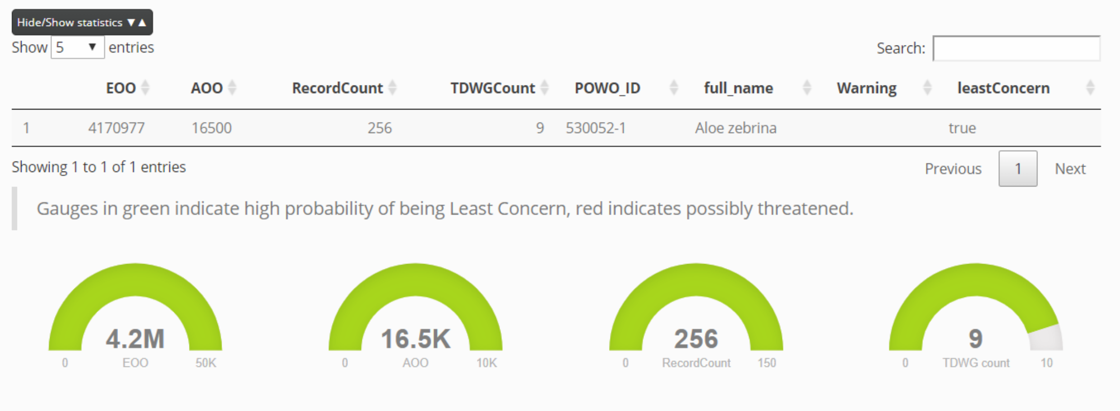

Statistics table and gauges to visualise whether LC thresholds have been met

SIS Tables and point file

Below the statistics table and gauges are a series of tables that provide the minimum information required to support a Least Concern Red List assessment.

The first tab shows the occurrence point table. This has been formatted to the IUCN spatial data standards.

Download the point file by clicking the **'Download clean point file'** button. Click the **'Download SIS Connect Files'** button to download the SIS connect files as a zip file. The following tables will be included in the download: *allfields*, *assessments*, *countries*, *credits*, *habitats*, *plantspecific*, *taxonomy* and all are linked through the *internal_taxon_id* field.

countries.csv. Note that the list of countries should follow the IUCN standard - see SIS Connect. We use the TDWG World Geographical Scheme for Recording Plant Distributions (WGSRPD) from POWO to determine the list of countries, but as there is not a one-to-one relationship with the IUCN standard, it is necessary to carefully examine the results to make sure all countries and subcountries are included before submission to SIS Connect.

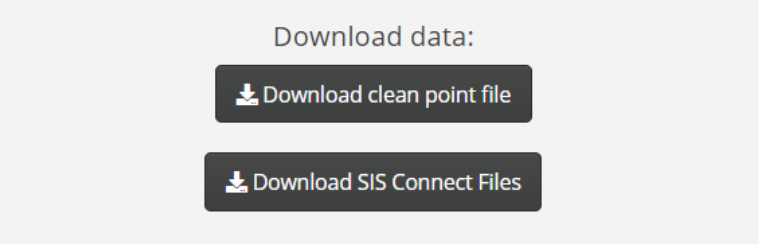

Download SIS connect files and point file

Click the **Clear form** button if you wish to reset the analysis

Additional data There are additional fields that can be entered e.g. **habitat** and **plant growth form** as well as assessor information. However, these fields can also be entered directly into the SIS database. Use the multiple select options from the sidebar on the left to pick the relevant habitat and growth form.

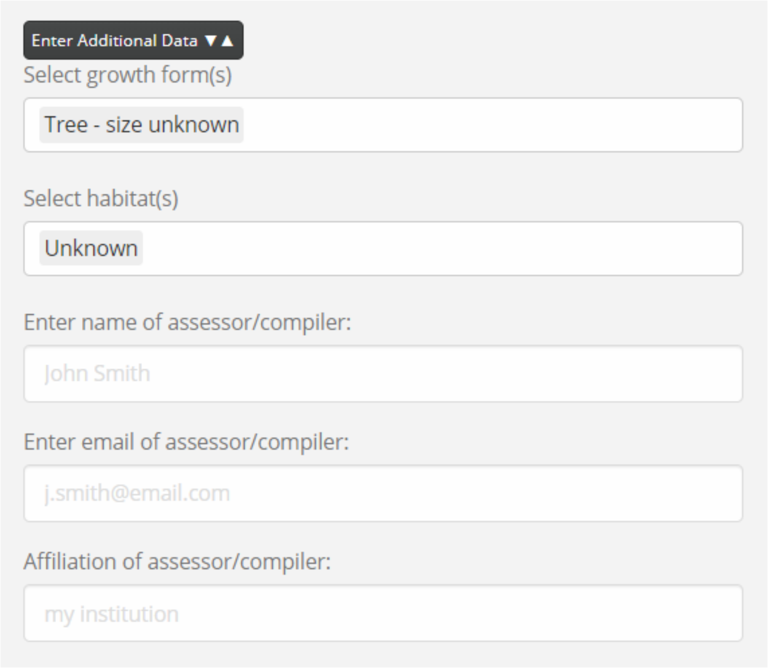

Enter additional data

For many species, the plant growth form can be found by querying the World Checklist of Selected Plant Families.

The lookup values for the **plantgrowthforms.plantgrowthformslookup** fields are:

Code	description
T	Tree - size unknown
TL	Tree - large
TS	Tree - small
S	Shrub - size unknown

SL	Shrub - large
SS	Shrub - small
F	Forb or Herb
A	Annual
GR	Graminoid
GE	Geophyte
V	Vines
H	Hydrophyte
P	Parasite
E	Epiphyte
L	Lithophyte
SC	Succulent - form unknown
SA	Succulent - annual
SH	Succulent - shrub
ST	Succulent - tree
PT	Fern
C	Cycad
M	Fungus
B	Moss
LC	Lichen

- **Habitat**. The habitat classification scheme can be found here: https://www.iucnredlist.org/resources/habitat-classification-scheme and additional descriptors here: https://nc.iucnredlist.org/redlist/content/attachment_files/dec_2012_guidance_habitats_classification_scheme.pdf

- **credits.csv**. The credits.csv file contains references to the people who helped to generate the assessment. Placeholder values have been added to the firstName, lastName, email and affiliation fields, but these must be filled in prior to submission. Please see the supporting information document for further details on crediting: https://www.iucnredlist.org/resources/supporting-information-guidelines

---

Batch

Try the quick start demo first:

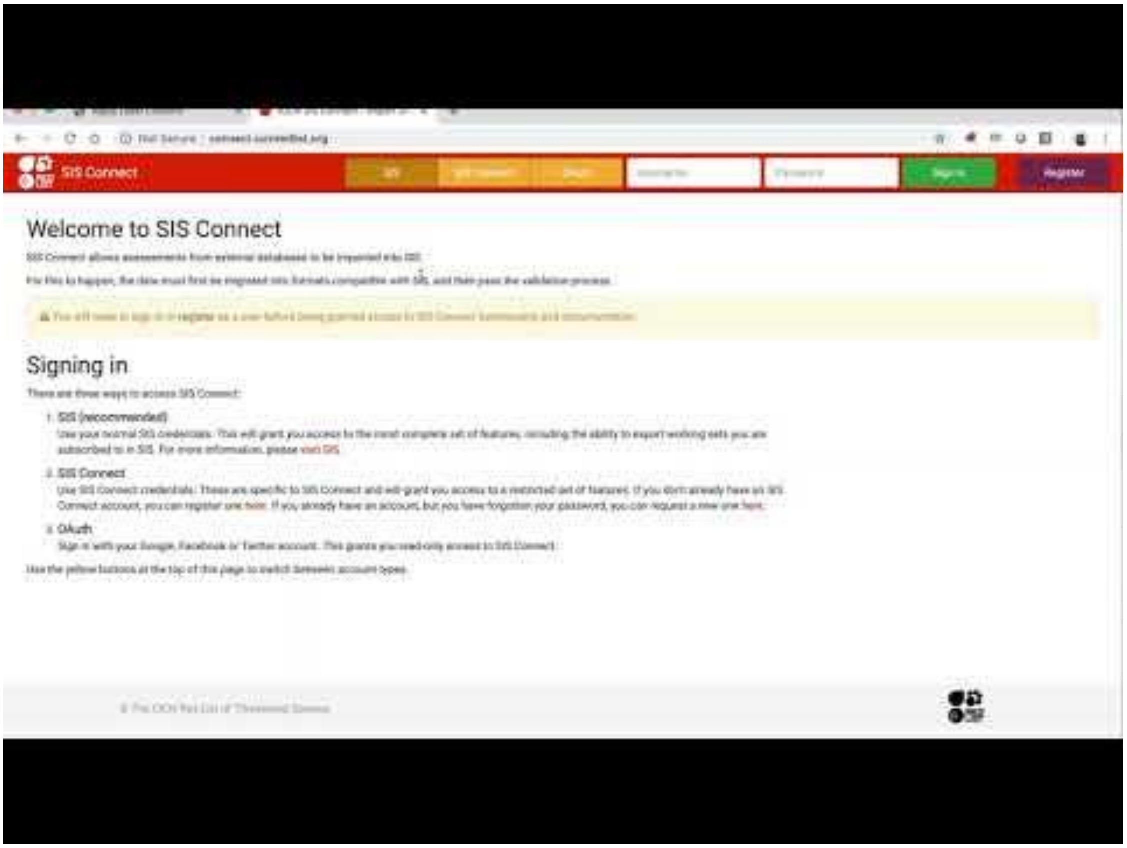

1 The batch option requires users to upload a list of names that can be processed together. The file needs to be a csv. with a *name_in* column. Click the **Browse…** button to upload the file. Depending on how many species are in your file, it may take a moment or two before the results table appears in the main panel. The names will have been checked against the POWO names backbone. The results table includes the *IPNI identifier*, *author* and *accepted status*, as well as the original *name_in* field.

The results show how many species were searched, how many names could not be matched, how many names matched to synonyms. Any names not matched, or matched to synonyms are highlighted in red and ommited from further calculations.

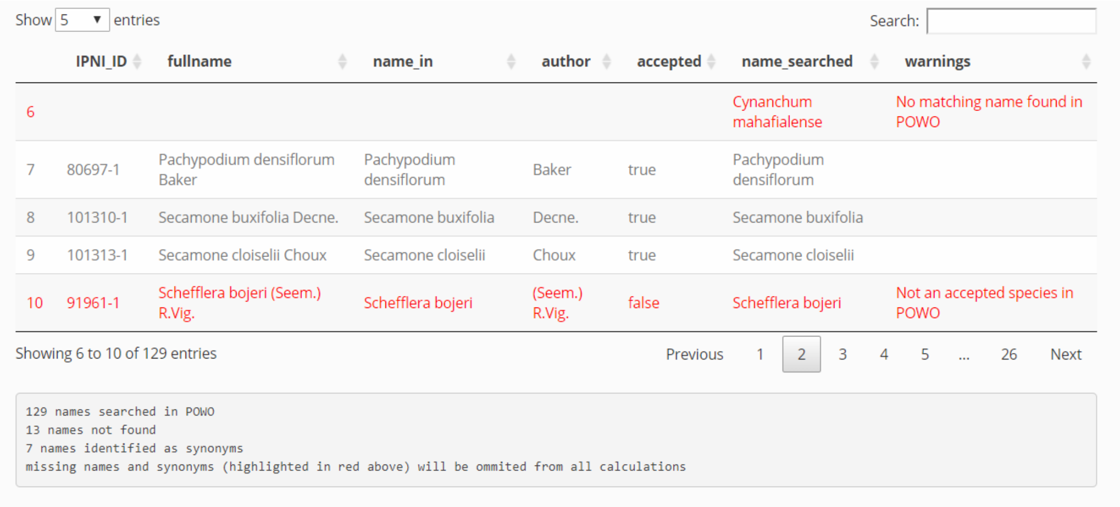

Show 5 entries Search:

	IPNI_ID	fullname	name_in	author	accepted	name_searched	warnings
6						Cynanchum mahafialense	No matching name found in POWO
7	80697-1	Pachypodium densiflorum Baker	Pachypodium densiflorum	Baker	true	Pachypodium densiflorum	
8	101310-1	Secamone buxifolia Decne.	Secamone buxifolia	Decne.	true	Secamone buxifolia	
9	101313-1	Secamone cloiselii Choux	Secamone cloiselii	Choux	true	Secamone cloiselii	
10	91961-1	Schefflera bojeri (Seem.) R.Vig.	Schefflera bojeri	(Seem.) R.Vig.	false	Schefflera bojeri	Not an accepted species in POWO

Showing 6 to 10 of 129 entries Previous 1 2 3 4 5 … 26 Next

```
129 names searched in POWO
13 names not found
7 names identified as synonyms
missing names and synonyms (highlighted in red above) will be ommited from all calculations
```

2 As with the single option, a limit can be set to the number of occurrence records to be downloaded from GBIF. The value can be set with the slider widget. We allow a maximum of 10,000 occurrence points, a minimum of 1,000 and the default is set at 3,000.

3 Click the Run Analysis! button to generate the raw statistics. As with the single process, the results contain the original search results fields (*IPNI identifier*, *author*, *accepted status* and *name_in*) and several metrics relating to geographic range size.

In contrast to the single process, the batch process allows the user to adjust the thresholds to determine Least Concern using the slider widgets. Species that meet or exceed the LC thresholds are highlighted in green.

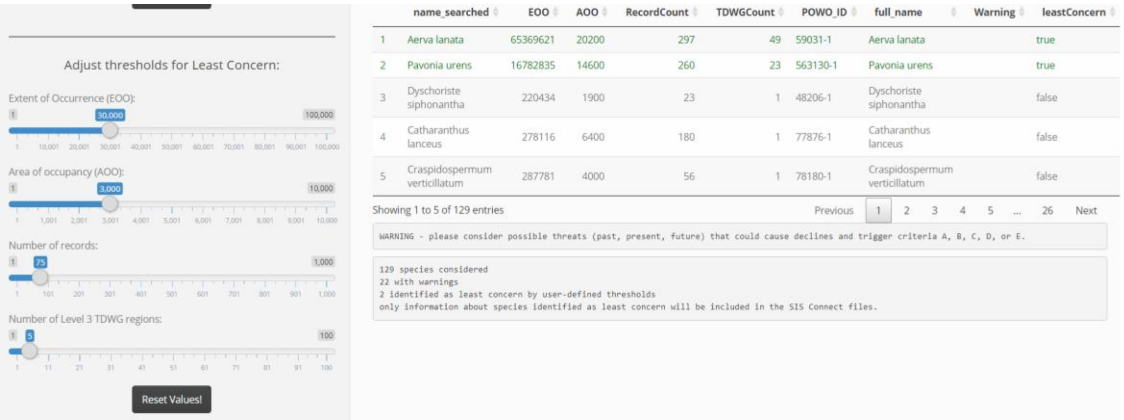

	name_searched	EOO	AOO	RecordCount	TDWGCount	POWO_ID	full_name	Warning	leastConcern
1	Aerva lanata	65369621	20200	297	49	59031-1	Aerva lanata		true
2	Pavonia urens	16782835	14600	260	23	563130-1	Pavonia urens		true
3	Dyschoriste siphonantha	220434	1900	23	1	48206-1	Dyschoriste siphonantha		false
4	Catharanthus lanceus	278116	6400	180	1	77876-1	Catharanthus lanceus		false
5	Craspidospermum verticillatum	287781	4000	56	1	78180-1	Craspidospermum verticillatum		false

Showing 1 to 5 of 129 entries Previous 1 2 3 4 5 … 26 Next

```
WARNING - please consider possible threats (past, present, future) that could cause declines and trigger criteria A, B, C, D, or E.
```

```
129 species considered
22 with warnings
2 identified as least concern by user-defined thresholds
only information about species identified as least concern will be included in the SIS Connect files.
```

Results of analysis

Below the table is a list of how many species were considered for the analysis and how many warnings there were i.e. species that could not be processed. Finally, the number of LC species identified is reported.

4 The user is asked to consider possible threats (past, present, future) that could cause declines and trigger criteria A, B, C, D, or E. If there are thought to be no other threats then the tick box can be checked to unlock the download button.

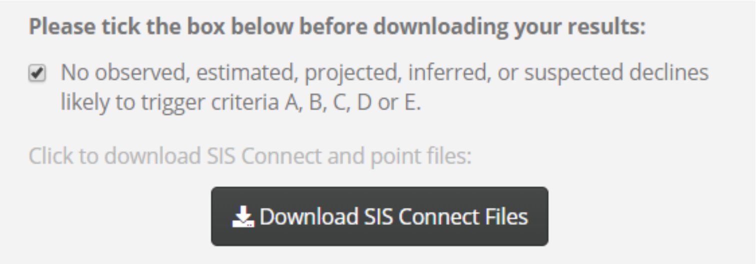

Download batch SIS Connect files

5 Click on the 'Download SIS Connect Files'.

The output files contain a line for each species, or multiple lines if there is a one-to-many relationship, and all files are linked through the unique identifier *internal_taxon_id*. The results.csv file is not required for the Red List assessment, but is provided for information. It contains the following fields: *name_searched*, *EOO*, *AOO*, *RecordCount*, *TDWGCount*, *POWO_ID*, *full_name*, *Warning*, *leastConcern*. The points.csv file contains all occurrence points in the native range and has been formatted to meet the IUCN mapping standards. The points.csv file is required to support the Red List assessment, but is not currently accepted through the SIS Connect system. All other files can be imported into SIS Connect.

Batch process with user points Some users may already have datasets with georeferenced and cleaned point data, so do not require a search of GBIF for occurrence records. User points can be included in the analysis by adding *latitude* and *longitude* fields to the input csv. file along with *name_in*. The batch procedure can then be followed as previously outlined.

What is missing? Some editing may be required prior to submitting the outputs to **SIS Connect**.

Next steps:

Review assessments (SGs and RLAs)

All Red List assessments need to be reviewed by a relevant Red List Authority. A list of plant Red List Authorities or Specialist Groups can be found here

Submit to SIS connect - needs login

When the csv. files have been updated with plant growth form, habitat and countries have been checked, the files can be uploaded to SIS Connect

Acknowledgements:

The following packages were used to create this app:

raster
here
magrittr

rgdal
DT
leaflet
rgbif
jsonlite tidyverse
httr
zip
shinythemes
wicket
sf
shiny
rCAT
flexdashboard
shinydashboard
shinyjs
